# Lexical Elaboration and Intentional Vocabulary Acquisition: An Eye-Tracking Study

**DOI:** 10.3390/jemr19030061

**Published:** 2026-06-01

**Authors:** Taegang Lee, Byeongchae Choi, Sungmook Choi, Soo-Ok Kweon

**Affiliations:** 1 Department of English Education, Kyungpook National University, Daegu 41566, Republic of Korea; taeganglee00@gmail.com (T.L.); rookie102412@knu.ac.kr (B.C.); sungmookchoi@knu.ac.kr (S.C.); 2 Division of Humanities and Social Sciences, Pohang University of Science and Technology, Pohang 37673, Republic of Korea

**Keywords:** lexical elaboration, eye tracking, vocabulary acquisition, input enhancement, learning context

## Abstract

Prior research on lexical elaboration (providing additional information about unfamiliar words) has yielded non-significant effects on incidental vocabulary acquisition. Building on this, the present study used eye-tracking technology to examine whether similar results replicate under intentional learning conditions. Participants were Korean undergraduate students (*N* = 67) who were randomly assigned to one of three groups: baseline, unenhanced elaboration, or enhanced elaboration. They were instructed to read an English text twice while their eye movements were recorded. After reading, they responded to two forewarned vocabulary assessments measuring form and meaning recall. Overall, the unenhanced elaboration group exhibited distinct target-word processing patterns and higher meaning recall performance compared to the baseline group, whereas the enhanced elaboration group’s outcomes were comparable to the baseline group. For instance, generalized linear mixed-effects modeling (GLMM) revealed that while the unenhanced elaboration group maintained stable reading times on target words across the two readings, the baseline and enhanced elaboration groups exhibited significant reductions. Furthermore, only the unenhanced elaboration group significantly outperformed the baseline group in meaning recall. Collectively, our findings suggest that lexical elaboration, particularly without input enhancement, may facilitate vocabulary acquisition under intentional learning conditions by promoting more sustained attention to unfamiliar words.

## 1. Introduction

In second language (L2) acquisition, lexical elaboration refers to the provision of additional information—such as definitions, synonyms, or appositions—that helps learners infer the meanings of unfamiliar words in a reading text [1]. Because it can make unfamiliar lexical items more accessible while preserving the informational richness of the text, lexical elaboration has received considerable attention in L2 reading research, particularly in relation to reading comprehension (e.g., [2,3]).

More recently, lexical elaboration has also been investigated as a potential source of vocabulary learning through reading. However, previous studies conducted in incidental learning contexts have generally failed to find clear benefits of lexical elaboration for vocabulary acquisition [1,4,5,6,7]. These findings suggest that simply providing additional lexical information may not be sufficient for learners to acquire unfamiliar word forms or establish form-meaning connections when their primary goal is to comprehend the text.

These findings suggest that, in incidental learning contexts, lexical elaboration alone may be insufficient to facilitate L2 vocabulary acquisition. A plausible explanation lies in learners’ allocation of attentional resources during reading. Because learners in incidental learning conditions typically read for overall comprehension rather than vocabulary learning, they may prioritize global text integration over the processing of unfamiliar lexical items and their corresponding elaborations [5,8]. This limited attentional engagement with novel words may constrain the depth of processing needed to establish stable form–meaning connections.

If the limited effects of lexical elaboration in incidental learning contexts can be attributed to learners’ allocation of attention, then a critical question is whether lexical elaboration may function differently in an intentional learning environment. In intentional conditions, learners are explicitly oriented toward vocabulary learning, typically through prior notice of a vocabulary test. This test expectation may encourage learners to allocate greater attention to unfamiliar words and their corresponding elaborations during reading, thereby reducing the attentional bottleneck that may constrain vocabulary acquisition in incidental contexts. However, whether lexical elaboration facilitates vocabulary acquisition under intentional learning conditions remains empirically untested.

Another unresolved issue concerns how learners process lexically elaborated input in real time. Previous studies have relied mainly on offline vocabulary and comprehension measures, leaving the attentional mechanisms underlying lexical elaboration largely unexplored. To address this gap, the present study uses eye-tracking to examine how lexical elaboration affects both the online processing and acquisition of unfamiliar words under intentional learning conditions, operationalized as reading under test expectation through forewarned vocabulary assessments. Specifically, we examine whether the provision of lexical elaboration leads learners to allocate more attention to target words during reading, as reflected in eye-movement measures, and whether such attention is associated with greater vocabulary gains.

The study also examines the role of visual input enhancement. Visually enhancing target word forms and their corresponding elaborations, for example, through boldfacing or underlining, may increase the salience of form–meaning relationships and direct learners’ attention to relevant lexical information. However, enhancement may also make these relationships more immediately accessible, potentially reducing the need for sustained inferential processing. Thus, the study compares unenhanced and enhanced lexical elaboration to determine whether visual enhancement provides any additional benefit for attention and vocabulary learning under intentional learning conditions.

Finally, the study explores whether lexical elaboration under intentional learning conditions entails a trade-off between vocabulary acquisition and reading comprehension. Because intentional learning directs learners’ attention to target lexical items, it may promote deeper lexical processing [9]. At the same time, this deliberate focus on vocabulary could divert attentional resources from global text integration, potentially compromising overall comprehension. This possible trade-off has not been directly examined in previous research on lexical elaboration.

The present study contributes to the literature in several ways. It extends prior research, which has primarily focused on incidental learning contexts, by examining lexical elaboration under intentional learning conditions. In doing so, it employs eye-tracking to investigate the underlying attentional processes during reading lexically elaborated texts, an aspect that has not been examined in previous studies (e.g., [1,4,6,7]). Additionally, by examining the potential trade-off between intentional vocabulary learning and reading comprehension, the study offers a more comprehensive account of the potential benefits and limitations of lexical elaboration. The following research questions guide this study:

1. To what extent do unenhanced and enhanced lexical elaborations influence the processing of unfamiliar words and their corresponding lexical elaborations, as measured by eye-tracking?
2. To what extent do unenhanced and enhanced lexical elaborations influence lexical gains, as measured by forewarned vocabulary assessments?
3. Do unenhanced and enhanced lexical elaborations result in a trade-off between vocabulary acquisition and reading comprehension?

## 2. Literature Review

### 2.1. Research on Lexical Elaboration on Incidental Vocabulary Learning

To date, five key studies [1,4,5,6,7] have examined the impact of lexical elaboration on vocabulary acquisition under incidental contexts. Notably, their findings consistently revealed no significant effects of lexical elaboration on either form acquisition or form–meaning mapping. Moreover, while only three of these studies assessed reading comprehension [1,4,6], no evidence of a trade-off between vocabulary acquisition and reading comprehension has been found.

For example, Chung [4] examined 484 Korean middle school students with low English proficiency. They read nine English passages—either unmodified or lexically elaborated—containing 20 target words. The elaborated passages included additional information (e.g., definitions, synonyms, antonyms) via apposition, signaling phrases, or coordination. The nine passages and a total of 20 multiple-choice comprehension questions were presented in a single test booklet. After completing all readings and comprehension questions, two unannounced vocabulary tests (form recognition and meaning recognition) were administered. Results revealed no significant differences in scores between those who read unmodified texts and those who read elaborated texts on the vocabulary tests or the reading comprehension test.

Similar results were reported by Urano [1], who examined lexical elaboration using individual sentences rather than a full text. Participants were 40 adult Japanese learners with varying levels of English proficiency. They were instructed to read 20 English sentences, 10 in unmodified form and 10 in elaborated form. In the elaborated sentences, synonyms of 20 target pseudowords were provided in apposition. After reading each sentence, participants answered a yes/no reading comprehension question. Following the completion of all sentences, two surprise vocabulary tests (form recognition and meaning recognition) were administered. The results showed no significant differences between the unmodified and elaborated conditions on any of the vocabulary or reading comprehension tests.

Building on these previous studies, Kim [5] explored the impact of lexical elaboration with and without input enhancement on incidental vocabulary acquisition but found no significant benefits in either the enhanced or unenhanced condition. In this study, 297 Korean undergraduates with intermediate English proficiency were assigned to one of three groups: (1) baseline, (2) unenhanced elaboration, and (3) enhanced elaboration. They read an English magazine article, corresponding to their assigned group, which included 26 target words. In the unenhanced version, additional information was provided through devices such as apposition and parallelism, while in the enhanced version, these target words were highlighted in bold (input enhancement). Immediately after reading, two unannounced vocabulary tests (form recognition and meaning recognition) were administered. The results indicated that lexical elaboration, with or without input enhancement, did not significantly improve performance on either test. Similar findings were reported by Chae and Lee [6], who likewise found no significant effects of lexical elaboration, regardless of input enhancement, on incidental vocabulary acquisition (measured by an unannounced meaning recognition test) and reading comprehension among 130 Korean high school students.

More recently, Park [7] examined whether repeated exposure to target words would enhance the effects of lexical elaboration on incidental vocabulary acquisition but failed to find such effects. A total of 100 university students read three passages focusing on meaning, either with or without lexical elaboration. Twelve target words were distributed across the passages and appeared four times each, with lexical elaboration provided only once at the first occurrence of each word. Following the reading, participants completed two unannounced vocabulary tests measuring meaning recognition and meaning recall. The results showed no significant differences between the elaborated and unelaborated conditions on either test.

### 2.2. Eye-Tracking in L2 Vocabulary Acquisition Research

In L2 reading research, a variety of methods have been employed to investigate how learners process specific linguistic features, such as unfamiliar vocabulary. These include offline techniques (e.g., note-taking, underlining, stimulated recall) and online techniques (e.g., think-aloud protocols, eye-tracking) [10]. Among these, eye-tracking technology, which records eye movements with millisecond precision using infrared cameras [11], has recently emerged as one of the most reliable tools for examining the real-time cognitive processing of linguistic input, particularly in the context of L2 vocabulary acquisition through reading.

This methodological preference stems from several advantages eye-tracking offers over traditional methods (e.g., underlining, think-aloud protocols). Specifically, it allows researchers to capture the moment-by-moment processing of unfamiliar words as learners encounter them in context, revealing how—and for how long—they engage with and revisit novel lexical items while reading (e.g., [12]). Moreover, as several scholars have noted (e.g., [12,13,14]), eye-tracking enables the examination of naturalistic lexical processing, as it unobtrusively registers readers’ eye movements without requiring secondary tasks often necessary in traditional techniques [15].

In examining lexical processing, researchers rely on specific eye-movement measures, such as saccades, regressions, and fixation count. Among these, total reading time (the cumulative duration of fixations within a region) and fixation count (the number of fixations on that region) have been most widely used in L2 vocabulary research to better understand how learners acquire unfamiliar words during reading (e.g., [16,17]). Studies to date have generally reported that longer reading times and more frequent fixations on unfamiliar words are associated with greater vocabulary gains (e.g., [15,18]). For instance, Choi [15] tracked the eye movements of 38 Korean undergraduates as they read an English text containing either visually enhanced or unenhanced target collocations. The results showed that students who read the visually enhanced text devoted more time to the target collocations and achieved higher scores on a collocation test than those who read the unenhanced text.

More recently, eye-tracking research has further shown that sustained reading time on a word across repeated encounters may be linked to greater learning gains (e.g., [19,20,21,22]). For instance, Koval [19] asked 40 U.S. undergraduate and graduate students to read 120 English sentences containing 24 Finnish target words. Each target word was presented four times, either in a massed condition (four consecutive exposures) or in a spaced condition (four exposures distributed at roughly 25-sentence intervals). Results showed that target-word processing time decreased more steeply in the massed condition than in the spaced condition, with the latter yielding significantly greater vocabulary gains.

Collectively, these findings indicate that eye-tracking provides a useful means of examining the attentional mechanisms underlying L2 vocabulary learning. However, in the context of lexical elaboration, such mechanisms have not been examined, thereby limiting clear a priori predictions while giving rise to competing possibilities. For instance, the presence of additional information (i.e., lexical elaborations) for unfamiliar words may draw L2 learners’ attention to such words or encourage more sustained processing across repeated encounters, potentially facilitating vocabulary acquisition. On the other hand, because lexical elaboration is likely to clarify word meanings, it may reduce the need for effortful meaning inference. This reduced processing effort may lead L2 learners to allocate less attention to unfamiliar words, potentially resulting in weaker learning outcomes. These competing possibilities may further vary depending on the presence of input enhancement, which could either increase attentional allocation by highlighting unfamiliar words and their corresponding lexical elaborations or reduce processing demands by making their relationships more immediately accessible. Exploring these possibilities is central to understanding the role of lexical elaboration in L2 vocabulary acquisition under intentional learning conditions, and the present study is, to our knowledge, the first to adopt eye-tracking technology to examine the role of lexical elaboration in attention allocation and intentional vocabulary learning.

## 3. Materials and Methods

### 3.1. Participants

The participants in this study were 67 Korean university students (26 males, 41 females; *M*_age_ = 22.64, *SD* = 2.29) with approximately 10 years of formal English instruction. The Nelson-Denny Reading Test (Form I) was administered to assess their English reading proficiency, yielding a mean score of 25.00 (*SD* = 5.18). This score is comparable to that of 9th-grade American students (see the NDRT I & J Examiner’s Manual), suggesting that the participants possessed a high-intermediate level of reading proficiency. Participants signed a consent form informing them that they would be required to read English texts while their eye movements were recorded, followed by several tests. As a token of appreciation for their participation, each participant received 30,000 KRW (approximately US $20). This study received approval from the Institutional Review Board (KNU 2023-12-26).

The participants were randomly assigned to one of three groups: the baseline group (*n* = 23), the unenhanced elaboration group (*n* = 22), and the enhanced elaboration group (*n* = 22). An analysis of the homogeneity of these three groups revealed no significant differences in English reading proficiency, as assessed by the Nelson-Denny Reading Test (*F*_2,64_ = 0.031, *p* = 0.970), or in age (*F*_2,64_ = 0.076, *p* = 0.927).

### 3.2. Materials

#### 3.2.1. Experimental Text and Target Words

Three versions of the experimental text were developed: baseline, unenhanced elaboration, and enhanced elaboration. The baseline version was adapted from The Boots of Hunain, a short story in World Tales [23]. The story depicted a conflict between a shoemaker and traveler attempting to buy boots, conveying a moral lesson on the dangers of arrogance. This story was chosen because its theme, commonly found in fables and moral tales, is widely recognizable and does not require substantial prior knowledge. Following Urano’s [1] methodology, the original story was modified to remove low-frequency words, ensuring that participants encountered only target words as unfamiliar vocabulary. The resulting baseline text contained six target words, 382 words in total, and 26 sentences, with a Flesch-Kincaid Grade Level of 5.6, indicating that a native English speaker with approximately six years of formal education should be able to comprehend it without difficulty. Given that the participants’ English reading proficiency was comparable to that of 9th-grade students in the United States, as assessed by Nelson-Denny Reading Test, it was assumed that they would have no difficulty understanding the text’s overall content.

**Figure 1:**
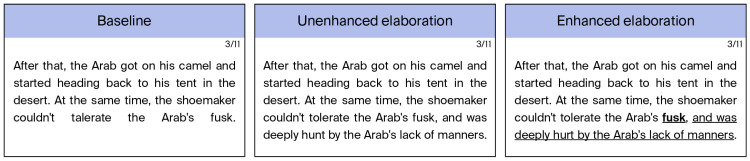
Examples of the Three Versions of the Experimental Text.

For the unenhanced elaboration version, lexical elaborations—defined as additional information that clarifies the meaning of target words (e.g., synonyms, restatements, clarifying phrases)—were added to the baseline version. In the enhanced elaboration version, both the target words and their lexical elaborations were underlined, and the target words were further bolded. Both the unenhanced and enhanced elaboration texts consisted of 430 words and 28 sentences. Figure 1 presents examples of the three versions of the experimental text, which served as the eye-tracking stimuli. The complete versions of the experimental text are provided in Appendix A, Appendix B and Appendix C.

To control for participants’ prior word knowledge, six pseudowords were embedded as learning targets in each version of the experimental text (see Table 1). These pseudowords were selected from Choi [24], who had sourced them from the ARC nonword database. This database provides pseudowords that conform to orthographic and phonotactic rules, ensuring that they contain valid bigrams [25]. Crucially, each target word (i.e., pseudoword) appeared once in the experimental text.

#### 3.2.2. Vocabulary Assessments: Form Recall and Meaning Recall Tests

To examine the effects of lexical elaboration on the acquisition of six target words, two vocabulary tests were administered: a form recall test and a meaning recall test. The form recall test assessed participants’ ability to recall the visual forms of the target words. In this test, participants were given a blank A4 sheet and asked to write down the forms of any unfamiliar words they recalled from the experimental text. The meaning recall test, by contrast, evaluated participants’ ability to associate the target words with their meanings. Participants received an A4 sheet displaying the target words’ forms and were instructed to write down their meanings either in Korean or in English.

#### 3.2.3. Reading Comprehension Test

A reading comprehension test was administered to examine the potential trade-off between vocabulary acquisition and overall comprehension under intentional vocabulary learning conditions. The test followed a cloze format, in which participants were provided with the experimental text containing 12 blanks for content words. The baseline group received the baseline text, whereas the unenhanced and enhanced elaboration groups were given the elaborated text, without any visual input enhancements.

### 3.3. Procedure

The experiment was conducted over two weeks. In the first week, participants visited the laboratory at their scheduled time and completed the 30 min Nelson-Denny Reading Test to assess their English reading proficiency. Approximately one week later, in the second week, participants returned to the laboratory and were seated approximately 60 cm away from an SMI-RED eye-tracker (Teltow, Germany). Eye-tracking calibration was performed using a five-point calibration method, where participants followed a moving red dot to ensure precise tracking of their eye movements throughout the experiment. After calibration, participants were instructed to read the experimental text at their own pace and were informed that a reading comprehension test and vocabulary tests would follow, thereby establishing an intentional learning condition through the expectation of a vocabulary test. The text was presented across 11 screens on the eye-tracking monitor, each displaying three to seven lines of text. The average number of words per screen was 34.73 for the baseline version and 39.10 for both the unenhanced and enhanced elaboration versions. Participants read their assigned version of the text twice, with no time limit, as repeated reading is a commonly used strategy among L2 learners to enhance vocabulary retention and comprehension. Following the second reading, participants completed a 10 min filler task, in which they read an English text unrelated to the experimental text. To ensure engagement with the task, they were informed that a reading comprehension test would follow. The filler task was designed to minimize the influence of short-term memory on subsequent test performance. After the filler task, participants took a 3 min reading comprehension test, followed by two vocabulary tests—form recall and meaning recall—each lasting one minute.

### 3.4. Analysis of Eye-Tracking Data

Eye-tracking data were extracted and analyzed using SMI BeGaze software (version 3.7, SensoMotoric Instruments). Following Kruger et al. [26], data from five participants with tracking ratios below the 80% threshold and one participant whose eye-tracking data contained excessive zero values were excluded, resulting in 61 participants for the final analysis. The primary region of interest (ROI) was the six target words, with their corresponding lexical elaborations used as an additional ROI. To examine participants’ eye movement within these ROIs, two eye-tracking metrics were utilized: total reading time and fixation count. ‘Total reading time’ refers to the cumulative duration of fixations on an ROI, while ‘fixation count’ denotes the total number of fixations on an ROI, both of which are widely used to examine the processing of unfamiliar lexical items in L2 reading research (e.g., [15]). In the present study, ‘total reading time’ served as the primary measure of learners’ processing of target words and their corresponding lexical elaborations.

### 3.5. Scoring

The two vocabulary tests were scored using lenient criteria. For the form recall test, one point was awarded if the spelling exactly matched or closely approximated the form of the target words (e.g., ‘tard’ for ‘tarb’). In the meaning recall test, one point was given if the meaning was correct or close enough not to distort the essential meaning of the target words (e.g., prit: ‘regret’ or ‘remorse’). A similar approach was applied to the reading comprehension test, where one point was awarded for the correct word, minor spelling errors (e.g., ‘chanse’ for ‘chance’), or a semantically similar response (e.g., ‘wise’ for ‘clever’).

### 3.6. Statistical Analysis

#### 3.6.1. Statistical Analysis of Eye-Tracking Data

All statistical analyses were conducted using R software (version 4.2.3; [27]). Before the main analysis, reading time for the first screen of the experimental text, which did not contain target words, was examined to determine whether the three groups differed in reading speed. No significant differences were found in either the first or second reading of the experimental text (all *p* values > 0.05), indicating that the three groups had comparable reading speeds.

The primary ROI was the six target words, with the lexical elaborations serving as an additional ROI. Both between-group and within-group analyses were conducted for the total reading time and fixation count of these ROIs. Shapiro–Wilk tests indicated that both eye-tracking metrics were non-normally distributed (all *p* values < 0.001), exhibiting positive skewness and zero values. The fixation count data also showed overdispersion, with its variance exceeding the mean. Accordingly, total reading time was analyzed using zero-inflated gamma generalized linear mixed-effects models (ZIG-GLMMs) to account for skewness and excess zeros [28], while fixation count was analyzed using zero-inflated negative binomial GLMMs (ZINB-GLMMs) to address overdispersion and zero inflation in discrete data [29,30].

Models were fitted using the glmmTMB function from the glmmTMB package [31]. To examine overall effects in target word processing, GLMMs were fitted with ‘group’ (three levels: baseline [intercept], unenhanced elaboration, and enhanced elaboration), ‘reading phase’ (two levels: first [intercept] and second readings of the experimental text), and their interaction as fixed effects. For lexical elaboration processing, the same model structure was used, except that ‘group’ had two levels (unenhanced elaboration and enhanced elaboration).

Follow-up between-group and within-group analyses were then conducted. For between-group analyses of target word processing, two models were fitted. In the first, the fixed effect was group with three levels: baseline (intercept), unenhanced elaboration, and enhanced elaboration. The second model included ‘group’ with two levels (unenhanced elaboration [intercept] and enhanced elaboration) to allow for direct comparison between the two elaboration conditions. For lexical elaboration processing, the fixed effect compared the unenhanced elaboration (intercept) and enhanced elaboration groups. For within-group analyses, two models were fitted—one for target word processing and one for lexical elaboration processing. In both models, reading phase (first and second readings) was included as the fixed effect, with the first reading serving as the intercept.

For all models described above, we first attempted to fit the maximal design-appropriate random-effects structure, including random intercepts for ‘participants’ and ‘target words’ and design-appropriate random slopes for predictors that varied within the corresponding random-effect unit, following Barr et al. [32]. When models with correlated random effects failed to converge, random correlations were removed while retaining the relevant random slopes where possible. If convergence problems persisted, the random-effects structure was simplified stepwise until a stable converged model was obtained, following common practice in mixed-effects modeling (e.g., [33,34]). The resulting final models therefore differed in their random-effects structures across analyses. The final fixed-effects and random-effects structures selected through this procedure are reported in Appendix D.

Model diagnostics were conducted using simulation-based residual analyses via the simulateResiduals function from the DHARMa package [35]. The results indicated no significant deviations from model assumptions, including uniformity, dispersion, and outliers across all models (all *p* values > 0.05), suggesting that the selected model distributions were appropriate. Additionally, model fit indices (*R*^2^ and ICC), computed using the r2 and icc functions from the performance package [36], yielded patterns consistent with model adequacy.

Fixed effects are reported as unstandardized regression coefficients (β), representing the predicted change in the dependent variable on a log scale, along with their standard errors (*SE*). Effect sizes for the eye-tracking analyses are reported as exp(β), representing the ratio of change in the eye-tracking measures.

#### 3.6.2. Statistical Analysis of Vocabulary Test Scores

For the vocabulary test data, GLMMs were fitted using the glmer function from the lme4 package [37], with the bobyqa optimizer and a maximum of 10^5^ function evaluations to facilitate model convergence. Separate models were constructed for form recall and meaning recall scores, with ‘group’—baseline (intercept), unenhanced elaboration, and enhanced elaboration—as the fixed effect. Random intercepts for ‘participants’ and ‘target words’ were included to account for individual and lexical variability. We also attempted to include a design-appropriate random slope for ‘group’, but these models did not converge; thus, random-intercept-only models were retained. The final model structures are reported in Appendix D. Post hoc comparisons were performed using the emmeans function from the emmeans package [38], applying the least significant difference (LSD) method. We adopted odds ratios (*OR*s; i.e., exponentiated coefficients) as the measure of effect size for the GLMMs, in accordance with Wang and Pellicer-Sánchez [39].

## 4. Results

### 4.1. Eye-Tracking Results

#### 4.1.1. Processing of Target Words

Descriptive statistics for target word processing are presented in Table 2. Generalized linear mixed-effects models including ‘group’, ‘reading phase’, and their interaction were fitted to examine overall patterns. As shown in Table 3, a significant interaction effect between ‘group’ and ‘reading phase’ was observed only for the unenhanced elaboration group, both for total reading time (β = 0.44, *SE* = 0.17, *p* = 0.012) and fixation count (β = 0.38, *SE* = 0.13, *p* = 0.004). In contrast, the corresponding interaction was not statistically significant for the enhanced elaboration group, either for total reading time (β = 0.25, *SE* = 0.17, *p* = 0.137) or fixation count (β = 0.21, *SE* = 0.13, *p* = 0.092). To further examine these effects, follow-up between-group and within-group analyses were conducted, as reported in the following sections.

#### Between-Group Analyses

Between-group comparisons showed no significant differences in total reading time or fixation count across the three groups (Table 4). For total reading time, no significant differences were found between the baseline and unenhanced elaboration groups (first reading: β = −0.13, *SE* = 0.15, *p* = 0.389, exp(β) = 0.88; second reading: β = 0.32, *SE* = 0.19, *p* = 0.091, exp(β) = 1.37), the baseline and enhanced elaboration groups (first reading: β = −0.10, *SE* = 0.14, *p* = 0.486, exp(β) = 0.91; second reading: β = 0.15, *SE* = 0.18, *p* = 0.404, exp(β) = 1.16), or the unenhanced and enhanced elaboration groups (first reading: β = −0.03, *SE* = 0.14, *p* = 0.848, exp(β) = 0.97; second reading: β = 0.17, *SE* = 0.18, *p* = 0.361, exp(β) = 1.18). Similar results were observed for fixation count. ZINB-GLMMs revealed no significant differences between the baseline and unenhanced elaboration groups (first reading: β = −0.05, *SE* = 0.13, *p* = 0.733, exp(β) = 0.96; second reading: β = 0.32, *SE* = 0.18, *p* = 0.073, exp(β) = 1.38), between the baseline and enhanced elaboration groups (first reading: β = 0.02, *SE* = 0.13, *p* = 0.897, exp(β) = 1.02; second reading: β = 0.23, *SE* = 0.17, *p* = 0.193, exp(β) = 1.25), or between the unenhanced and enhanced elaboration groups (first reading: β = −0.06, *SE* = 0.13, *p* = 0.633, exp(β) = 0.94; second reading: β = 0.10, *SE* = 0.17, *p* = 0.576, exp(β) = 1.10).

#### Within-Group Analyses

The results showed that the baseline and enhanced elaboration groups exhibited significant reductions in total reading time and fixation count from the first to the second reading of the experimental text, whereas the unenhanced elaboration group did not (Table 5). In the baseline group, both total reading time (β = −0.61, *SE* = 0.12, *p* < 0.001, exp(β) = 0.54) and fixation count (β = −0.48, *SE* = 0.13, *p* < 0.001, exp(β) = 0.62) significantly decreased from the first to the second reading. In contrast, the unenhanced elaboration group showed no significant differences in either total reading time (β = −0.17, *SE* = 0.12, *p* = 0.149, exp(β) = 0.84) or fixation count (β = −0.16, *SE* = 0.12, *p* = 0.173, exp(β) = 0.85). For the enhanced elaboration group, significant reductions were observed in total reading time (β = −0.34, *SE* =0.12, *p* = 0.005, exp(β) = 0.71) and fixation count (β = −0.29, *SE* = 0.12, *p* = 0.016, exp(β) = 0.74).

#### 4.1.2. Processing of Lexical Elaborations

Descriptive statistics for the processing of lexical elaborations are presented in Table 6. GLMM analyses revealed no significant interaction effect between ‘group’ and ‘reading phase’ (see Table 7). Specifically, the interaction effect for the enhanced elaboration group was not statistically significant in either total reading time (β = −0.14, *SE* = 0.11, *p* = 0.197) or fixation count (β = −0.15, *SE* = 0.10, *p* = 0.144). To further examine potential patterns, follow-up between-group and within-group analyses were conducted and are reported in the following sections.

#### Between-Group Analyses

As shown in Table 8, the results revealed no significant differences in total reading time between the groups, regardless of reading phase (first reading: β = 0.03, *SE* = 0.16, *p* = 0.837, exp(β) = 1.03; second reading: β = −0.11, *SE* = 0.17, *p* = 0.494, exp(β) = 0.89). Similarly, fixation count analyses showed no significant differences between the two groups during either the first (β = 0.02, *SE* = 0.12, *p* = 0.843, exp(β) = 1.03) or the second reading (β = −0.10, *SE* = 0.15, *p* = 0.478, exp(β) = 0.90) of the experimental text.

#### Within-Group Analyses

As presented in Table 9, a series of GLMMs revealed that the unenhanced elaboration group did not exhibit significant decreases in either total reading time (β = −0.12, *SE* = 0.09, *p* = 0.174, exp(β) = 0.88) or fixation count (β = −0.10, *SE* = 0.07, *p* = 0.126, exp(β) = 0.88). In contrast, the enhanced elaboration group demonstrated significant reductions in both measures from the first to the second reading (total reading time: β = −0.27, *SE* = 0.07, *p* < 0.001, exp(β) = 0.77; fixation count: β = −0.27, *SE* = 0.05, *p* < 0.001, exp(β) = 0.74).

### 4.2. Results of Form Recall and Meaning Recall Tests

Table 10 presents descriptive statistics for two vocabulary tests (form recall and meaning recall). In the form recall test, the enhanced elaboration group achieved the highest scores (*M* = 1.68, *SD* = 1.49), followed by the unenhanced elaboration (*M* = 1.25, *SD* = 1.16), and baseline (*M* = 1.00, *SD* = 0.86) groups.

GLMM results (Table 11) indicated no statistically significant difference between the baseline and unenhanced elaboration groups (β = 0.25, *SE* = 0.42, *p* = 0.558, *OR* = 1.28). Similarly, the comparison between the baseline and enhanced elaboration groups did not reveal a significant difference (β = 0.74, *SE* = 0.40, *p* = 0.063, *OR* = 2.10). The difference between the unenhanced and enhanced elaboration groups was also not significant (β = −0.50, *SE* = 0.40, *p* = 0.212, *OR* = 0.61).

In the meaning recall test, the unenhanced elaboration group achieved the highest scores (*M* = 1.35, *SD* = 1.31), followed by the enhanced elaboration (*M* = 0.77, *SD* = 0.75), and baseline (*M* = 0.55, *SD* = 0.83) groups. Results from GLMM analysis revealed a significant difference between the baseline and unenhanced elaboration groups (β = 1.20, *SE* = 0.48, *p* = 0.012, *OR* = 3.31). In contrast, the difference between the baseline and enhanced elaboration groups was not statistically significant (β = 0.43, *SE* = 0.49, *p* = 0.374, *OR* = 1.54). Similarly, the difference between the unenhanced and enhanced elaboration groups did not reach statistical significance (β = 0.76, *SE* = 0.43, *p* = 0.076, *OR* = 2.15).

### 4.3. Reading Comprehension Test Results

The results of the reading comprehension test showed that the unenhanced elaboration group achieved the highest scores (*M* = 6.25, *SD* = 2.65), followed by the enhanced elaboration group (*M* = 6.14, *SD* = 2.03), and the baseline group (*M* = 5.15, *SD* = 2.64). A Shapiro–Wilk test confirmed that the data met the assumption of normality (*p* > 0.05). Accordingly, a one-way ANOVA was performed, which revealed no significant difference in scores among the three groups (*F*_2,59_ = 1.237, *p* = 0.298).

## 5. Discussion

Utilizing eye-tracking technology, this study examined the effects of lexical elaboration on the processing and acquisition of unfamiliar L2 words under intentional learning conditions. Overall, the results suggest an advantage of lexical elaboration for form-meaning mapping under intentional learning conditions, particularly in the unenhanced condition. Specifically, (Section 4.1.1 and Table 3, Table 4 and Table 5), the unenhanced elaboration group maintained consistent processing times for target words across repeated readings and significantly outperformed the baseline group in meaning recall (though not in form recall) (Section 4.2 and Table 11). In contrast, the enhanced elaboration group showed a significant reduction in processing time for target words (Table 5) and did not significantly outperform the baseline group in either form or meaning recall (Section 4.2 and Table 11)**.** Additionally, no trade-off was observed between vocabulary acquisition and reading comprehension (Section 4.3). In the following, these findings are discussed in detail in relation to the three research questions.

The first research question asked: To what extent does the use of unenhanced and enhanced lexical elaborations influence the processing (measured via eye-tracking) of unfamiliar words and their corresponding lexical elaborations? Interestingly, while between-group analyses revealed no significant differences across the three groups (baseline, unenhanced elaboration, and enhanced elaboration) (Table 4), within-group comparisons indicated a distinct target-word processing pattern for the unenhanced elaboration group across repeated readings (Table 5)**.** For instance, ZIG-GLMM analyses revealed that only the unenhanced elaboration group maintained consistent target-word processing times across the two readings, whereas both the baseline and enhanced elaboration groups demonstrated significant reductions (Table 5)**.** This pattern is consistent with the significant group × reading phase interaction for the unenhanced elaboration group, which was observed for both total reading time and fixation count (see Table 3). Notably, as will be discussed later, the distinct reading pattern of the unenhanced elaboration group may have contributed to their superior performance in the meaning recall test (Section 4.2 and Table 11).

Given the absence of previous eye-tracking research on lexical elaboration, interpreting the present results remains challenging. Nonetheless, the notable within-group differences observed in the current study (Table 5) may tentatively be interpreted as reflecting differences in reading strategies. For instance, in the baseline group, the absence of lexical elaborations may have led to shallow, superficial processing—such as lexical guessing with limited contextual support—leading to less sustained processing of the target words for inferring or verifying their meanings across readings. In contrast, the enhanced elaboration group may have adopted a different reading strategy, establishing rapid form–meaning mappings during the first reading by directly connecting the visually salient target words and elaborations. Consequently, the need for additional inference or verification of target word meanings during the second reading may have been reduced.

The unenhanced elaboration group, however, did not have visually salient cues to signal target-word meanings. As a result, they may have engaged in ongoing processes of inferring, verifying, and refining the meanings of target words across repeated readings, as reflected in their stable processing times for both target words (Table 5) and their lexical elaborations (Table 9)**.** It should be noted that these interpretations remain speculative and should not be taken as direct evidence of underlying cognitive processing, thus warranting further investigation into this processing. Nevertheless, the present study contributes to the existing body of literature (e.g., [1,5]) as the first study to utilize eye-tracking technology to explore the role of lexical elaboration in L2 vocabulary acquisition.

The second research question was: To what extent does the use of unenhanced and enhanced lexical elaborations influence lexical gains (measured via forewarned vocabulary assessments)? For form recall, GLMM analyses showed no statistically significant differences across the groups (Section 4.2 and Table 11)**,** indicating that lexical elaboration, regardless of input enhancement, may not benefit the acquisition of orthographic forms of unfamiliar words. This finding is consistent with previous studies conducted under incidental learning conditions [1,4,5] and extends them by demonstrating that the null effect of lexical elaboration on word form acquisition also holds under the intentional learning conditions. Collectively, these findings suggest that lexical elaboration, whether enhanced or not, may not facilitate the acquisition of unfamiliar word forms in intentional and incidental learning contexts examined in the present and previous studies (e.g., [1,4,7]).

In contrast, the meaning recall results revealed a significant advantage for the unenhanced elaboration group over the baseline group (Section 4.2 and Table 11)**,** indicating that lexical elaboration may facilitate form–meaning connections for unfamiliar words. This finding diverges from previous research conducted under incidental learning contexts [1,4,5,6,7], which reported no such benefit on either meaning recognition or meaning recall. This discrepancy may be attributed to the intentional learning context adopted in the present study, where participants were informed in advance of the subsequent vocabulary test. This study therefore extends previous research (e.g., [1,5]) which was conducted under incidental learning contexts, while providing novel evidence that lexical elaboration, without input enhancement, may provide an advantage for form-meaning mapping under the present intentional learning conditions.

Interestingly, the meaning recall results showed no significant difference between the baseline and enhanced elaboration groups (Section 4.2 and Table 11), consistent with previous research (e.g., [5]). This finding is intriguing because the visual salience in the enhanced elaboration condition, which likely highlighted the connection between target words and their corresponding elaborations, did not appear to provide additional benefits beyond lexical elaboration alone for meaning recall. Collectively, the findings from the present study and previous studies [5,6] suggest that lexical elaboration with input enhancement may not provide clear advantages for form-meaning connections in both incidental and intentional learning contexts examined in these studies.

Taken together with the eye-tracking results, the differential effects of lexical elaboration with and without input enhancement on meaning recall may be linked to the groups’ distinct reading patterns across repeated readings. This interpretation is supported by the comprehensive model (Table 3), which showed a significant group × reading phase interaction for the unenhanced elaboration group, but not for the enhanced elaboration group, as well as by the within-group analyses (Table 5 and Table 9). Specifically, the eye-tracking data revealed that the unenhanced elaboration group devoted comparable processing time to target words and their corresponding elaborations across readings (Table 5 and Table 9)**,** which may be indicative of more sustained engagement with target words. This sustained engagement may be associated with their superior meaning-recall performance (Section 4.2 and Table 11)**,** consistent with broader linguistic findings that prolonged lexical processing across multiple exposures facilitates greater vocabulary learning (e.g., [19,20,21,22]). In contrast, the enhanced elaboration group may have bypassed the need to sustain their engagement, as the visual salience of input enhancement likely made the connection between target words and their associated elaborations immediately apparent. Consequently, such salience may have encouraged more superficial processing of target words, without providing clear benefits for form-meaning mapping.

The third research question was: Do unenhanced and enhanced lexical elaborations result in a trade-off between vocabulary acquisition and reading comprehension? The results revealed no significant difference in reading comprehension score among the three groups, indicating the absence of such a trade-off across both the unenhanced elaboration and enhanced elaboration groups. Though not directly comparable, this finding aligns with previous studies conducted under incidental vocabulary learning contexts [1,4,6], while extending them by showing that even under intentional vocabulary learning contexts, where learners are guided to devote more time to processing target words, lexical elaboration may not compromise overall comprehension. Taken together with the vocabulary test results (Section 4.2 and Table 11)**,** the finding suggests that lexical elaboration, when presented without input enhancement, may facilitate vocabulary form–meaning mappings while maintaining reading comprehension under intentional learning conditions.

Despite the novel findings reported above, this study is subject to several limitations that should be considered when interpreting its findings. First, the study employed a relatively short experimental text (approximately 400 words) and included only a single text. Second, only six target words were used. Although the GLMM analysis included target words as a random effect to account for item-level variability, the limited number of items and the use of a single text may restrict the generalizability of the findings and may partly reflect the specific characteristics of the experimental text and lexical elaborations used in the study. Accordingly, the present results should be interpreted with caution, and future research should examine whether the current findings can be replicated using longer texts, multiple passages, and a larger set of target items. Third, each target word appeared only once during reading. Since repeated exposure plays a crucial role in vocabulary acquisition (e.g., [40]), future studies might examine how lexical elaboration interacts with various exposure frequencies. Fourth, although the pseudowords in this study conformed to orthographic and phonotactic rules, their unfamiliar grapheme-phoneme correspondences may have posed processing challenges [16], potentially affecting participants’ form recall and meaning recall scores (Section 4.2 and Table 10)**.** Fifth, reading comprehension in the present study was assessed using a cloze-test format. Although this format has been widely used to measure reading comprehension (e.g., [41,42]), it may rely more on local processing than on global text understanding. This limitation should be considered when interpreting the absence of a trade-off between vocabulary learning and reading comprehension. Future research may employ alternative formats (e.g., multiple-choice tests) to further validate this finding. Sixth, in the present study, intentional learning was operationalized as learning under the expectation of a vocabulary test. As the task involved repeated reading, the findings should be interpreted within this specific task configuration, and further research is needed to determine whether they generalize to other intentional learning contexts. Finally, all lexical elaborations in this study were presented after target words. However, the effectiveness of lexical elaboration may depend on its placement. When lexical elaborations precede unfamiliar words, they may preactivate word meanings, reducing the need for inference and leading to shallower processing. In contrast, lexical elaborations appearing after unfamiliar words may encourage deeper processing by prompting learners to actively resolve meaning, potentially strengthening form–meaning mappings. Given the lack of prior research on this issue, future studies might investigate how the effects of lexical elaboration on the processing and subsequent learning of unfamiliar words vary according to the placement of lexical elaborations.

## 6. Conclusions

Using eye-tracking technology, this study explored the efficacy of lexical elaboration within intentional vocabulary learning contexts. The findings suggest that lexical elaboration may be associated with improved vocabulary acquisition under intentional learning contexts without negatively impacting reading comprehension. Interestingly, however, the strategy of visually enhancing target word forms and their corresponding elaborations did not appear to prompt learners to allocate additional attention to this information, which may be linked to the absence of significant lexical gains. These results, combined with previous research, suggest that the effectiveness of lexical elaboration may vary across learning contexts (incidental vs. intentional) and may also depend on the nature of the input (with or without visual enhancement). By examining lexical elaboration in an intentional learning setting, this study provides exploratory evidence that may broaden the scope of L2 acquisition research on lexical elaboration beyond incidental learning contexts.

Future studies could extend the present findings by comparing learners who are informed in advance of a vocabulary test with those who read without such test expectation. This comparison would help clarify the extent to which awareness of an upcoming test influences eye-movement patterns, attention to target words and lexical elaborations, and vocabulary learning outcomes.

## Figures and Tables

**Table 1:** Target Words, Meanings, and Lexical Elaborations.

Target Words	Meanings	Lexical Elaborations
fusk	rudeness	and was deeply hurt by the Arab’s lack of manners.
gond	tired	or tired
lomb	vanish	completely missing
prit	regret	a deep sense of regret bothering him because he had the chance to take the first shoe before but didn’t.
sult	impracticality	I mean, what could he do with just one shoe, right?
tarb	angry	with strong anger

Note. Complete experimental texts containing target words and their corresponding lexical elaborations are provided in Appendix B and Appendix C.

**Table 2:** Descriptive Statistics for Processing of Target Words (*SD* in Parentheses).

	1st Reading	1st Reading	2nd Reading	2nd Reading
Group	Total Reading Time	Fixation Count	Total Reading Time	Fixation Count
Baseline	1144.76 (527.21)	5.03 (1.88)	615.54 (311.55)	3.17 (1.40)
Unenhanced elaboration	1023.75 (423.57)	4.99 (2.15)	876.19 (508.06)	4.41 (2.61)
Enhanced elaboration	1071.96 (486.40)	5.21 (1.97)	852.45 (731.92)	4.26 (2.51)

Note. Total reading time is measured in milliseconds.

**Table 3:** Results of the Generalized Linear Mixed-Effects Models for Target Word Processing.

	Total Reading Time	Total Reading Time	Fixation Count	Fixation Count
Fixed Effects	β (*SE*)	*p*	β (*SE*)	*p*
(Intercept)	7.01 (0.12)	<0.001	1.56 (0.13)	<0.001
Unenhanced elaboration	−0.13 (0.15)	0.388	−0.07 (0.14)	0.635
Enhanced elaboration	−0.10 (0.14)	0.494	0.01 (0.14)	0.933
Reading phase	−0.60 (0.12)	<0.001	−0.47 (0.10)	<0.001
Unenhanced elaboration × reading phase	0.44 (0.17)	0.012	0.38 (0.13)	0.004
Enhanced elaboration × reading phase	0.25 (0.17)	0.137	0.21 (0.13)	0.092

**Table 4:** Between-Group Comparisons of the Processing of Target Words.

	1st Reading	1st Reading	1st Reading	1st Reading	2nd Reading	2nd Reading	2nd Reading	2nd Reading
	Total Reading Time	Total Reading Time	Fixation Count	Fixation Count	Total Reading Time	Total Reading Time	Fixation Count	Fixation Count
Fixed Effects	β (*SE*)	*p*	β (*SE*)	*p*	β (*SE*)	*p*	β (*SE*)	*p*
(Intercept)	7.00 (0.13)	<0.001	1.56 (0.13)	<0.001	6.41 (0.14)	<0.001	1.05 (0.15)	<0.001
Unenhanced elaboration	−0.13 (0.15)	0.389	−0.05 (0.13)	0.733	0.32 (0.19)	0.091	0.32 (0.18)	0.073
Enhanced elaboration	−0.10 (0.14)	0.486	0.02 (0.13)	0.897	0.15 (0.18)	0.404	0.23 (0.17)	0.193
Unenhanced elaboration vs. enhanced elaboration	−0.03 (0.14)	0.848	−0.06 (0.13)	0.633	0.17 (0.18)	0.361	0.10 (0.17)	0.576

**Table 5:** Within-Group Comparisons of the Processing of Target Words.

		Total Reading Time	Total Reading Time	Fixation Count	Fixation Count
Group	Fixed Effects	β (*SE*)	*p*	β (*SE*)	*p*
Baseline	(Intercept)	7.01 (0.12)	<0.001	1.57 (0.13)	<0.001
Baseline	2nd reading	−0.61 (0.12)	<0.001	−0.48 (0.13)	0.001
Unenhanced elaboration	(Intercept)	6.89 (0.12)	<0.001	1.51 (0.13)	<0.001
Unenhanced elaboration	2nd reading	−0.17 (0.12)	0.149	−0.16 (0.12)	0.173
Enhanced elaboration	(Intercept)	6.90 (0.12)	<0.001	1.58 (0.12)	<0.001
Enhanced elaboration	2nd reading	−0.34 (0.12)	0.005	−0.29 (0.12)	0.016

**Table 6:** Descriptive Statistics for the Processing of Lexical Elaborations (*SD* in Parentheses).

	1st Reading	1st Reading	2nd Reading	2nd Reading
Group	Total Reading Time	Fixation Count	Total Reading Time	Fixation Count
Unenhanced elaboration	3234.93 (1395.94)	18.15 (7.71)	2824.55 (1860.22)	15.93 (9.88)
Enhanced elaboration	3110.53 (1438.24)	17.39 (6.16)	2236.60 (1051.01)	12.82 (4.44)

Note. Total reading time is measured in milliseconds.

**Table 7:** Results of the Generalized Linear Mixed-Effects Models for Lexical Elaboration Processing.

	Total Reading Time	Total Reading Time	Fixation Count	Fixation Count
Fixed Effects	β (*SE*)	*p*	β (*SE*)	*p*
(Intercept)	7.73 (0.28)	<0.001	2.55 (0.28)	<0.001
Enhanced elaboration	0.03 (0.16)	0.859	0.04 (0.12)	0.772
Reading phase	−0.12 (0.08)	0.129	−0.13 (0.07)	0.089
Enhanced elaboration × reading phase	−0.14 (0.11)	0.197	−0.15 (0.10)	0.144

**Table 8:** Between-Group Comparisons of the Processing of Lexical Elaborations.

	1st Reading	1st Reading	1st Reading	1st Reading	2nd Reading	2nd Reading	2nd Reading	2nd Reading
	Total Reading Time	Total Reading Time	Fixation Count	Fixation Count	Total Reading Time	Total Reading Time	Fixation Count	Fixation Count
Fixed Effects	β (*SE*)	*p*	β (*SE*)	*p*	β (*SE*)	*p*	β (*SE*)	*p*
(Intercept)	7.72 (0.30)	<0.001	2.55 (0.30)	<0.001	7.61 (0.26)	<0.001	2.44 (0.27)	<0.001
Enhanced elaboration	0.03 (0.16)	0.837	0.02 (0.12)	0.843	−0.11 (0.17)	0.494	−0.10 (0.15)	0.478

**Table 9:** Within-Group Comparisons of the Processing of Lexical Elaborations.

		Total Reading Time	Total Reading Time	Fixation Count	Fixation Count
Group	Fixed Effects	β (*SE*)	*p*	β (*SE*)	*p*
Unenhanced elaboration	(Intercept)	7.72 (0.30)	<0.001	2.53 (0.31)	<0.001
Unenhanced elaboration	2nd reading	−0.12 (0.09)	0.174	−0.10 (0.07)	0.126
Enhanced elaboration	(Intercept)	7.76 (0.26)	<0.001	2.60 (0.26)	<0.001
Enhanced elaboration	2nd reading	−0.27 (0.07)	<0.001	−0.27 (0.05)	<0.001

**Table 10:** Descriptive Statistics for Form Recall and Meaning Recall Tests (*SD* in Parentheses).

Group	Form Recall Scores	Meaning Recall Scores
Baseline	1.00 (0.86)	0.55 (0.83)
Unenhanced elaboration	1.25 (1.16)	1.35 (1.31)
Enhanced elaboration	1.68 (1.49)	0.77 (0.75)

Note. The maximum possible score for both tests was 6.

**Table 11:** GLMM Results for Form and Meaning Recall Scores.

	Form Recall	Form Recall	Meaning Recall	Meaning Recall
Fixed Effects	β (*SE*)	*p*	β (*SE*)	*p*
(Intercept)	−1.86 (0.39)	<0.001	−2.66 (0.50)	<0.001
Unenhanced elaboration	0.25 (0.42)	0.558	1.20 (0.48)	0.012
Enhanced elaboration	0.74 (0.40)	0.063	0.43 (0.49)	0.374
Unenhanced elaboration vs. enhanced elaboration	−0.50 (0.40)	0.212	0.76 (0.43)	0.076

## Data Availability

The original contributions presented in this study are included in the article. Further inquiries can be directed to the corresponding author.

## Appendix A

### Baseline Version of the Experimental Text

Once upon a time, there was an Arab man who lived far from the town. One day, he rode his camel into town and found a pair of shoes for sale in a shop. These shoes really caught his eye, so he went into the shop and tried to negotiate a better price with the person who made the shoes. Unfortunately, the shoemaker refused to lower the price. This left the Arab man feeling very tarb. He shouted out loudly, “The price is as high as my camel!” After that, the Arab got on his camel and started heading back to his tent in the desert. At the same time, the shoemaker couldn’t tolerate the Arab’s fusk. The clever shoemaker, who knew where the Arab was from and which way he would go, took the shoes and went to a place the Arab would pass by. There, he strategically placed one shoe on the soft desert sand and dropped the other one far away. With his plan ready, he hid and patiently waited to see what would happen next. Later, the Arab noticed the first shoe lying on the sand. He said to himself, “That’s one of Shoemaker’s shoes. If only there were another shoe, I could have a complete pair without spending a single coin.” So, he continued on his path, recognizing the sult of having just one shoe in the desert. After a short while, the Arab came across the second shoe on his way to his tent. He felt prit for not collecting the first shoe earlier. Now, he really wanted a matching pair. This strong desire pushed him to return for the first shoe. To make sure his camel didn’t get too gond during this return journey, he got off and secured it with a rope. Then, he quickly went back to get the first shoe. Just at that moment, the shoemaker seized his chance. He came out of hiding and stole the Arab’s camel. When the Arab returned to the spot where he had left his camel, he discovered it had lombed. Believing it must have wandered away, he headed back to his people’s tents with a heavy heart. When his friend asked him what he had brought back from town, he replied sadly, “Only Shoemaker’s shoes.”

## Appendix B

### Unenhanced Elaboration Version of the Experimental Text

Once upon a time, there was an Arab man who lived far from the town. One day, he rode his camel into town and found a pair of shoes for sale in a shop. These shoes really caught his eye, so he went into the shop and tried to negotiate a better price with the person who made the shoes. Unfortunately, the shoemaker refused to lower the price. This left the Arab man feeling very tarb. With strong anger, he shouted out loudly, “The price is as high as my camel!” After that, the Arab got on his camel and started heading back to his tent in the desert. At the same time, the shoemaker couldn’t tolerate the Arab’s fusk, and was deeply hurt by the Arab’s lack of manners. The clever shoemaker, who knew where the Arab was from and which way he would go, took the shoes and went to a place the Arab would pass by. There, he strategically placed one shoe on the soft desert sand and dropped the other one far away. With his plan ready, he hid and patiently waited to see what would happen next. Later, the Arab noticed the first shoe lying on the sand. He said to himself, “That’s one of Shoemaker’s shoes. If only there were another shoe, I could have a complete pair without spending a single coin.” So, he continued on his path, recognizing the sult of having just one shoe in the desert. I mean, what could he do with just one shoe, right? After a short while, the Arab came across the second shoe on his way to his tent. He felt prit for not collecting the first shoe earlier, a deep sense of regret bothering him because he had the chance to take the first shoe before but didn’t. Now, he really wanted a matching pair. This strong desire pushed him to return for the first shoe. To make sure his camel didn’t get too gond or tired during this return journey, he got off and secured it with a rope. Then, he quickly went back to get the first shoe. Just at that moment, the shoemaker seized his chance. He came out of hiding and stole the Arab’s camel. When the Arab returned to the spot where he had left his camel, he discovered it had lombed, completely missing. Believing it must have wandered away, he headed back to his people’s tents with a heavy heart. When his friend asked him what he had brought back from town, he replied sadly, “Only Shoemaker’s shoes.”

## Appendix C

### Enhanced Elaboration Version of the Experimental Text

Once upon a time, there was an Arab man who lived far from the town. One day, he rode his camel into town and found a pair of shoes for sale in a shop. These shoes really caught his eye, so he went into the shop and tried to negotiate a better price with the person who made the shoes. Unfortunately, the shoemaker refused to lower the price. This left the Arab man feeling very **tarb**. With strong anger, he shouted out loudly, “The price is as high as my camel!” After that, the Arab got on his camel and started heading back to his tent in the desert. At the same time, the shoemaker couldn’t tolerate the Arab’s **fusk**, and was deeply hurt by the Arab’s lack of manners. The clever shoemaker, who knew where the Arab was from and which way he would go, took the shoes and went to a place the Arab would pass by. There, he strategically placed one shoe on the soft desert sand and dropped the other one far away. With his plan ready, he hid and patiently waited to see what would happen next. Later, the Arab noticed the first shoe lying on the sand. He said to himself, “That’s one of Shoemaker’s shoes. If only there were another shoe, I could have a complete pair without spending a single coin.” So, he continued on his path, recognizing the **sult** of having just one shoe in the desert. I mean, what could he do with just one shoe, right? After a short while, the Arab came across the second shoe on his way to his tent. He felt **prit** for not collecting the first shoe earlier, a deep sense of regret bothering him because he had the chance to take the first shoe before but didn’t. Now, he really wanted a matching pair. This strong desire pushed him to return for the first shoe. To make sure his camel didn’t get too **gond** or tired during this return journey, he got off and secured it with a rope. Then, he quickly went back to get the first shoe. Just at that moment, the shoemaker seized his chance. He came out of hiding and stole the Arab’s camel. When the Arab returned to the spot where he had left his camel, he discovered it had **lombed**, completely missing. Believing it must have wandered away, he headed back to his people’s tents with a heavy heart. When his friend asked him what he had brought back from town, he replied sadly, “Only Shoemaker’s shoes.”

## Appendix D

### Final Fixed-Effects and Random-Effects Structures for GLMM Analyses

**Analysis (ROI)**	**Dependent Variable**	**Fixed Effects**	**R** **andom Effects**
Comprehensive eye-tracking analysis (target words)	Total reading time	Group × Reading phase	(1|Participant) + (0 + Reading phase|Participant) + (1|Item)
Comprehensive eye-tracking analysis (target words)	Fixation count	Group × Reading phase	(1|Participant) + (1|Item)
Comprehensive eye-tracking analysis (lexical elaborations)	Total reading time	Group × Reading phase	(1|Participant) + (0 + Reading phase|Participant) + (1|Item)
Comprehensive eye-tracking analysis (lexical elaborations)	Fixation count	Group × Reading phase	(1|Participant) + (0 + Reading phase|Participant) + (1|Item)
Between-group eye-tracking analysis (target words)	Total reading time	Group	(1|Participant) + (1|Item)
Between-group eye-tracking analysis (target words)	Fixation count	Group	(1|Participant) + (1|Item)
Between-group eye-tracking analysis (lexical elaborations)	Total reading time	Group	(1|Participant) + (1|Item)
Between-group eye-tracking analysis (lexical elaborations)	Fixation count	Group	(1|Participant) + (1|Item)
Within-group eye-tracking analysis—baseline group (target words)	Total reading time	Reading phase	(1|Participant) + (0 + Reading phase|Participant) + (1|Item)
Within-group eye-tracking analysis—baseline group (target words)	Fixation count	Reading phase	(1|Participant) + (0 + Reading phase|Participant) + (1|Item)
Within-group eye-tracking analysis—unenhanced elaboration group (target words)	Total reading time	Reading phase	(1|Participant) + (0 + Reading phase|Participant) + (1|Item)
Within-group eye-tracking analysis—unenhanced elaboration group (target words)	Fixation count	Reading phase	(1|Participant) + (0 + Reading phase|Participant) + (1|Item)
Within-group eye-tracking analysis—enhanced elaboration group (target words)	Total reading time	Reading phase	(1|Participant) + (0 + Reading phase|Participant) + (1|Item)
Within-group eye-tracking analysis—enhanced elaboration group (target words)	Fixation count	Reading phase	(1|Participant) + (0 + Reading phase|Participant) + (1|Item)
Within-group eye-tracking analysis—unenhanced elaboration group (lexical elaborations)	Total reading time	Reading phase	(1|Participant) + (1|Item)
Within-group eye-tracking analysis—unenhanced elaboration group (lexical elaborations)	Fixation count	Reading phase	(1|Participant) + (0 + Reading phase|Participant) + (1|Item)
Within-group eye-tracking analysis—enhanced elaboration group (lexical elaborations)	Total reading time	Reading phase	(1|Participant) + (1|Item)
Within-group eye-tracking analysis—enhanced elaboration group (lexical elaborations)	Fixation count	Reading phase	(1|Participant) + (1|Item)
Vocabulary test analysis	Form recall scores	Group	(1|Participant) + (1|Item)
Vocabulary test analysis	Meaning recall scores	Group	(1|Participant) + (1|Item)
Note. Random-effects structures are presented using lme4-style notation.			
